# Double-gate structure enabling remote Coulomb scattering-free transport in atomic-layer-deposited IGO thin-film transistors with HfO_2_ gate dielectric through insertion of SiO_2_ interlayer

**DOI:** 10.1038/s41598-024-58330-1

**Published:** 2024-04-01

**Authors:** Cheol Hee Choi, Taikyu Kim, Min Jae Kim, Gwang-Bok Kim, Jeong Eun Oh, Jae Kyeong Jeong

**Affiliations:** 1 Department of Electronic Engineering, Hanyang University, Seoul, 04763 Republic of Korea; 2 Electronic Materials Research Center, Korea Institute of Science and Technology, Seoul, 02792 Republic of Korea

**Keywords:** Electrical and electronic engineering, Electronic devices

## Abstract

In this paper, high-performance indium gallium oxide (IGO) thin-film transistor (TFT) with a double-gate (DG) structure was developed using an atomic layer deposition route. The device consisting of 10-nm-thick IGO channel and 2/48-nm-thick SiO_2_/HfO_2_ dielectric was designed to be suitable for a display backplane in augmented and virtual reality applications. The fabricated DG TFTs exhibit outstanding device performances with field-effect mobility (*μ*_FE_) of 65.1 ± 2.3 cm^2^V^−1^ s^−1^, subthreshold swing of 65 ± 1 mVdec^−1^, and threshold voltage (*V*_TH_) of 0.42 ± 0.05 V. Both the (*μ*_FE_) and *SS* are considerably improved by more than two-fold in the DG IGO TFTs compared to single-gate (SG) IGO TFTs. Important finding was that the DG mode of IGO TFTs exhibits the nearly temperature independent *μ*_FE_ variations in contrast to the SG mode which suffers from the severe remote Coulomb scattering. The rationale for this disparity is discussed in detail based on the potential distribution along the vertical direction using technology computer-aided design simulation. Furthermore, the DG IGO TFTs exhibit a greatly improved reliability with negligible *V*_TH_ shift of − 0.22 V under a harsh negative bias thermal and illumination stress condition with an electric field of − 2 MVcm^−1^ and blue light illumination at 80 °C for 3600 s. It could be attributed to the increased electrostatic potential that results in fast re-trapping of the electrons generated by the light-induced ionization of deep level oxygen vacancy defects.

## Introduction

Amorphous oxide semiconductors (AOSs), such as indium–gallium–zinc–oxide (IGZO) and indium–gallium–zinc–tin–oxide (IGZTO), are widely used in display backplane technology for large-area active matrix-liquid crystal display (AMLCD) and -organic light emitting diodes (AMOLED) due to the remarkable electrical characteristics, such as reasonable field-effect mobility (*µ*_FE_) of > 10 cm^2^V^−1^ s^−1^, ultralow off-currents of < 10^−18^ Aμm^−1^, and steep switching characteristics^1–12^. However, the emerging augmented and virtual reality (AR/VR) headsets requiring display backplanes with ultrahigh resolution (≥ 2000 ppi) are fabricated using Si CMOS backplanes unlike the traditional flat panel displays, such as mobile and television, where the AOS thin-film transistors (TFTs) have been successfully implemented^13^. To achieve such an ultrahigh resolution using the AOS TFTs, the facile integration process and architecture of submicron scale AOS TFTs should be developed on the glass substrate. Furthermore, their lower on-current (*I*_ON_) compared to Si transistors makes it more challenging to utilize the AOS TFTs for AR/VR applications.

In this context, several approaches have been proposed to ensure high mobility AOS TFTs, such as heterojunction structures using quasi-two-dimensional electron gas (q2DEG)^5,11^, crystallization^10,12^, hydrogen doping^7^, and multi-gate architecture^14–19^. Amongst, adopting the multi-gate architecture, such as double-gate (DG), tri-gate and gate-all-around (GAA), is considered promising due to the outstanding current boosting ability. In practice, Mativenga et al. reported that IGZO TFTs with DG structure reveal seven times higher *I*_ON_ than those with single gate (SG) structure due to the bulk accumulation^15^. In addition to the current boosting, it was also confirmed that the DG structure greatly enhances a reliability for positive gate-bias thermal stress (PBTS) duration^16^. More importantly, the multi-gate structure is highly advantageous for overcoming short-channel effects (SCEs). Note that the natural length (*λ*) which determines the minimum gate length can be described as follows^19,20^:

1 $$\lambda = \sqrt{\frac{{\varepsilon }_{{\text{ch}}}{t}_{{\text{ch}}}{t}_{{\text{OX}}}}{N{\varepsilon }_{{\text{OX}}}}}$$

where *t*_ch_, *t*_ox_, *ε*_ch_, *ε*_ox_, and *N* are the thickness and dielectric constants of the bulk channel and dielectric layer, and the effective gate number, respectively. As such, the *λ* can be reduced by increasing not only the dielectric permittivity (*κ*) but also the *N*. For these reasons, the multi-gate structure as well as the high-*κ* gate dielectric have been adopted for the continual scaling down of Si transistors in the semiconductor industry^8^. Likewise, the AOS TFT based on them should be intensively investigated for its diverse potential applications.

In this study, high-performance IGO TFTs with the DG structure were developed using atomic layer deposition (ALD). Mostly, previous AOS TFTs with the DG structure have been fabricated using the sputtering method for the channel layer deposition. However, it is unsuitable for the three-dimensional (3D) muti-gate architecture due to its poor step coverage^21,22^. In contrast, the ALD-derived gate/channel stack employed in this work offers excellent step coverage and thickness controllability, which is highly suitable for the 3D structure such as GAA and channel-all-around (CAA) etc^23,24^. The choice of 10-nm-thick IGO as a channel layer is due to its low effective electron mass and high *µ*_FE_^10^. 2-/48-nm-thick high-*κ* SiO_2_/HfO_2_ dielectric films were used as a gate dielectric layer. That is, the *λ* was designed to be approximately 12.3 nm, which is expected to allow for a short channel length of ≤ 100 nm without the noticeable SCEs^25^. An important finding in this study is that the IGO TFTs with the DG structure exhibit the remote Coulomb scattering (RCS)-free transporting mechanism unlike those with the SG structure where the RCS and polar phonon scattering significantly occur^26^. This disparity can be explained by the concept of bulk accumulation, which was demonstrated through technology computer-aided design (TCAD) simulation. This phenomenon helps the Fermi-level (*E*_F_) reach the conduction band edge (*E*_CB_) rapidly, greatly improving both the *µ*_FE_ and subthreshold swing (*SS*) in the DG TFTs. Finally, the photo-bias stability of DG IGO TFTs was found to be superior to that of SG IGO TFTs even under negative bias thermal and illumination stress (NBTIS), which could be attributed to the increased electrostatic potential by the DG structure which greatly promotes the re-trapping of electrons originating from light illumination-induced oxygen vacancy (V_O_) ionization.

## Methods

### Preparation of semiconducting and dielectrics films

#### Semiconducting oxide film

10-nm-thick IGO thin-films as a channel layer were grown by plasma-enhanced ALD (PEALD) (NexusBe Co. Ltd.). The liquid metal precursors [3-(dimethylamino)propry]-dimethyl indium (DATI) and trimethyl gallium (TMG) were used as the In and Ga precursor, respectively. The DATI canister was heated to approximately 80 °C to provide a sufficient vapor pressure while the TMG canister was kept at room temperature. Each precursor was injected into the source line where high-purity Ar gas (99.999%) was used as a carrier gas for precursor delivery. O_2_ plasma was used as an oxidant, which was provided by applying an electric field (plasma power = 150 W) to the Ar/O_2_ mixed gas.

#### Dielectric film

Hafnium oxide (HfO_2_) and silicon oxide (SiO_2_) films were deposited by the PEALD (iSAC Research, South Korea). The metal precursors used for Hf and Si were diisopro-pylamino silane (DIPAS) and tetrakis ethylmethylamino hafnium (TEMAHf), respectively. The TEMAHf canister was heated to approximately 110 °C to provide a sufficient vapor pressure while the DIPAS canister was kept at room temperature. Likewise, each precursor was carried and purged by high-purity Ar gas.

### Film characterizations

The thickness of channel and dielectric films was measured using spectroscopic ellipsometry (Ellipso Technology Co.). The chemical properties of channel and dielectric films were examined through depth-profiling of X-ray photoelectron spectroscopy (XPS) (K-Alpha + , Thermo Fisher Scientific Co.) with an X-ray source of monochromatic Al K_*α*_ at 1486.6 eV.

### Device characterizations

**Figure 1:**
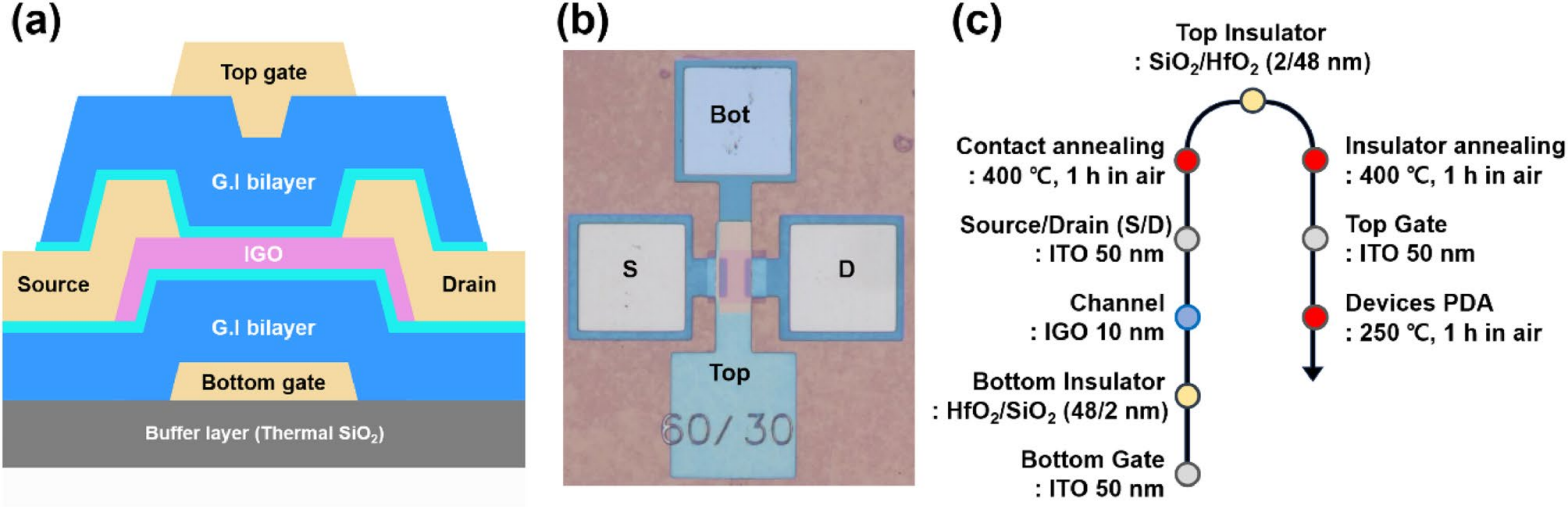
(**a**) A cross-sectional schematic of a DG IGO TFT. (**b**) A false colored top view image. (**c**) The device fabrication procedure.

The DG IGO TFTs were fabricated on thermally grown SiO_2_/Si substrate. 50-nm-thick indium-in oxide (ITO) films were deposited by DC magnetron sputtering at room temperature as bottom gate (BG) electrodes. The cation composition ratio is 9:1 (In:Sn). The BG electrodes were patterned through a conventional photolithography and wet etching process. Then, 2-/48-nm-thick SiO_2_/HfO_2_ gate dielectric stacks were grown by the PEALD at 250 °C. Note that the ultrathin SiO_2_ acts as an interfacial stabilizer. Subsequently, 10-nm-thick IGO thin-films were deposited by PEALD at 150 °C, followed by patterning using the photolithography and wet etching process. Next, 50-nm-thick ITO was deposited as source/drain (S/D) electrodes. The S/D electrodes were patterned by the standard photolithography with wet etching process, followed by postdeposition annealing (PDA) at 400 °C in ambient air for 1 h. The fabricated TFTs have the channel width (*W*) and length (*L*) of 60 and 30 µm, respectively. To fabricated the DG TFTs, the same gate dielectric stacks were deposited onto the underlying BG TFTs and annealed at 400 °C in ambient air for 1 h. Contact holes were formed by reactive ion etching (RIE). Top gate (TG) electrodes were deposited and patterned using the same method with the BG electrodes. Finally, the fabricated TFTs were annealed at 250 °C in ambient air for 1 h. Figure 1 shows a cross-sectional device schematic, an optical top view image, and the entire fabrication process.

## Results and discussion

### Electrical properties of DG IGO TFTs

**Figure 2:**
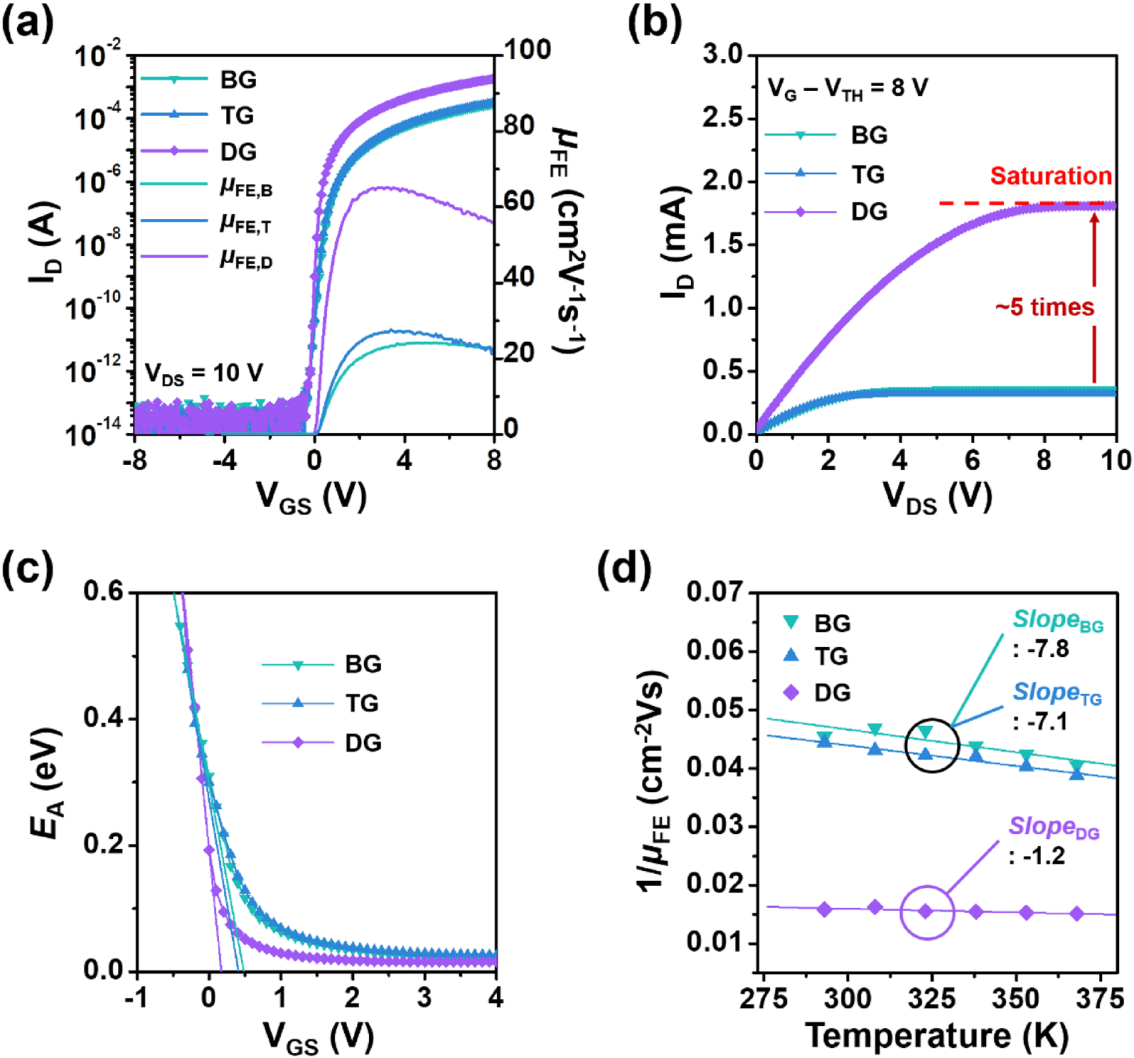
Sweeping gate electrode-dependent electrical characteristics: (**a**) transfer characteristics at V_DS_ = 10 V; (**b**) output characteristics; (**c**) variation of *E*_A_; and (**d**) 1/*µ*_FE_-*T* characteristics.

Electrical performances of IGO TFTs were evaluated using transfer characteristics at drain-to-source voltage (*V*_DS_) of 10 V. Note that the *μ*_FE_ under a saturation region was calculated using the following equation:

2 $${\mu }_{FE}={\left(\frac{\partial \sqrt{{I}_{D}}}{{\partial V}_{GS}}\right)}^{2}\frac{2L}{W{C}_{OX}}$$

where *I*_D_, *V*_GS_, and *C*_ox_ are the drain current, the gate voltage, and, the dielectric capacitance per unit area, respectively. The *V*_TH_ and *SS* were determined using linear extrapolation of *I*_D_^0.5^ versus *V*_GS_ (see Figure S1) and the equation *SS* = *dV*_GS_/*d*log(*I*_D_), respectively. Before covering the electrical characteristics, it should be noted that TG (BG) mode means that the input gate voltage is applied to the top (bottom) gate with a floating state of bottom (top) gate. DG mode denotes that the top gate is electrically wired to the bottom gate. BG IGO TFTs with HfO_2_ gate dielectric (48 nm) have the reasonable *µ*_FE_ of 18.1 ± 1 cm^2^V^−1^ s^−1^, *SS* of 130 ± 5 mVdec^−1^, *V*_TH_ of 0.22 ± 0.2 V and current modulation ratio (*I*_ON/OFF_) of ~ 10^9^ (Figure S2a and Table S1). It sufferes from the clock-wise hysteresis of 0.3 V, indicating the free electron carrier trapping into the HfO_2_ dielectric. Insertion of 2-nm-thick SiO_2_ interfacial layer between the IGO and HfO_2_ films greatly mitigates the operational hysteresis (0.05 V) in the resulting IGO TFTs (Figure S2b). Simultaneously, the *µ*_FE_ and *SS* are improved to 24.7 ± 0.7 cm^2^V^−1^ s^−1^ and 110 ± 5 mVdec^−1^, respectively, while the comparable *I*_ON/OFF_ is maintained (also see Table S1). TG IGO TFTs have almost the same device performances with the reasonable *µ*_FE_ of 25.8 ± 0.5 cm^2^V^−1^ s^−1^, *SS* of ~ 134 ± 5 mVdec^−1^, *V*_TH_ of 0.82 ± 0.1 V and *I*_ON/OFF_ ratio of ~ 10^10^ (Fig. 2a and Table 1). Meanwhile, DG IGO TFTs exhibit significantly improved device performances with *µ*_FE_ of 65.2 ± 2.3 cm^2^V^−1^ s^−1^, *SS* of 65 ± 1 mVdec^−1^, *V*_TH_ of 0.42 ± 0.05 V, and *I*_ON/OFF_ of ~ 10^10^. The enhancement in the *µ*_FE_ can be explained by a bulk accumulation conduction mechanism, which could occur when the depth of band bending is larger than half of the channel thickness (*t*_ch_)^15^. The greatly improved *SS* in the DG mode is attributed to the increased electrostatic potential enabling the *E*_F_ to rapidly rise toward the *E*_CB_, which can be experimentally demonstrated through the *V*_GS_-dependent change of activation energy (*E*_A_) (Fig. 2c): the falling rate (*F*_R_), defined as ǀΔ*E*_A_/Δ*V*_GS_ǀ, was extracted to 1.13 eVV^−1^ in the DG mode, which is twice as high as the SG mode (~ 0.6 eVV^−1^). This higher *F*_R_ also indicates fast transition from trap-limited conduction to percolation conduction. This improved electron transport can be also observed in the output characteristics, which have a fivefold higher *I*_D_ than the SG mode (Fig. 2b). The detailed output characteristics can be seen in Figure S3.

**Table 1:** Summary of electrical figures of merit of the IGO TFTs with different driving gate electrodes.

	BG	TG	DG
*μ*_FE_ [cm^2^V^−1^ s^−1^]	24.5 ± 0.8	25.8 ± 0.5	65.2 ± 2.3
*SS* [mVdec^−1^]	146 ± 5	134 ± 5	65 ± 1
*V*_TH_ [V]	0.94 ± 0.1	0.82 ± 0.1	0.42 ± 0.05
*I*_ON/OFF_	~ 10^10^	~ 10^10^	~ 10^11^
*I*_D_ @ *V*_GS_ − *V*_TH_ = 8 V [mA]	0.38	0.33	1.85
Falling rate [eVV^−1^]	0.62	0.67	1.13
Slope value	− 7.8	− 7.1	− 1.2

One thing we should identify is which scattering mechanism, phonon scattering or RCS, dominantly affects the electron transport in the IGO TFTs with SiO_2_/HfO_2_ gate dielectrics. The dominant scattering mode can be distinguished by the differential function for temperature (*T*) of Matthiessen’s rule as follows:

3 $$d\left(\frac{1}{{\mu }_{FE}}\right)/dT=\beta \alpha {T}^{\alpha -1}-\frac{\gamma }{{T}^{2}}$$

where *α*, *β*, and *γ* are the positive constants independent of *T*. A negative value in the 1/*µ*_FE_-*T* curve indicates that the RCS is dominant^26^. The larger absolute values, the greater scattering effect. SG IGO TFTs with the 48-nm-thick HfO_2_ gate dielectric have the high negative slope of − 15.4 (see Figure S2b and Table S1). It indicates the dominance of RCS, which is greatly mitigated by inserting 2-nm-thick SiO_2_. The extracted slope values are − 7.1 ~  − 7.8 in the TG and BG IGO TFTs with the 2-/48-nm-thick SiO_2_/HfO_2_, respectively (Fig. 2d). More importantly, it is further reduced down to − 1.2 in the DG TFTs, which implies that the RCS effect revealed in the SG TFTs (either BG or TG) greatly diminishes (Fig. 2d and Table 1). That is, it could be possible that the RCS-free zone is formed and contributes to the improvements in the electrical characteristics in the DG IGO TFTs.

### Chemical and dielectric properties of SiO_2_/HfO_2_

**Figure 3:**
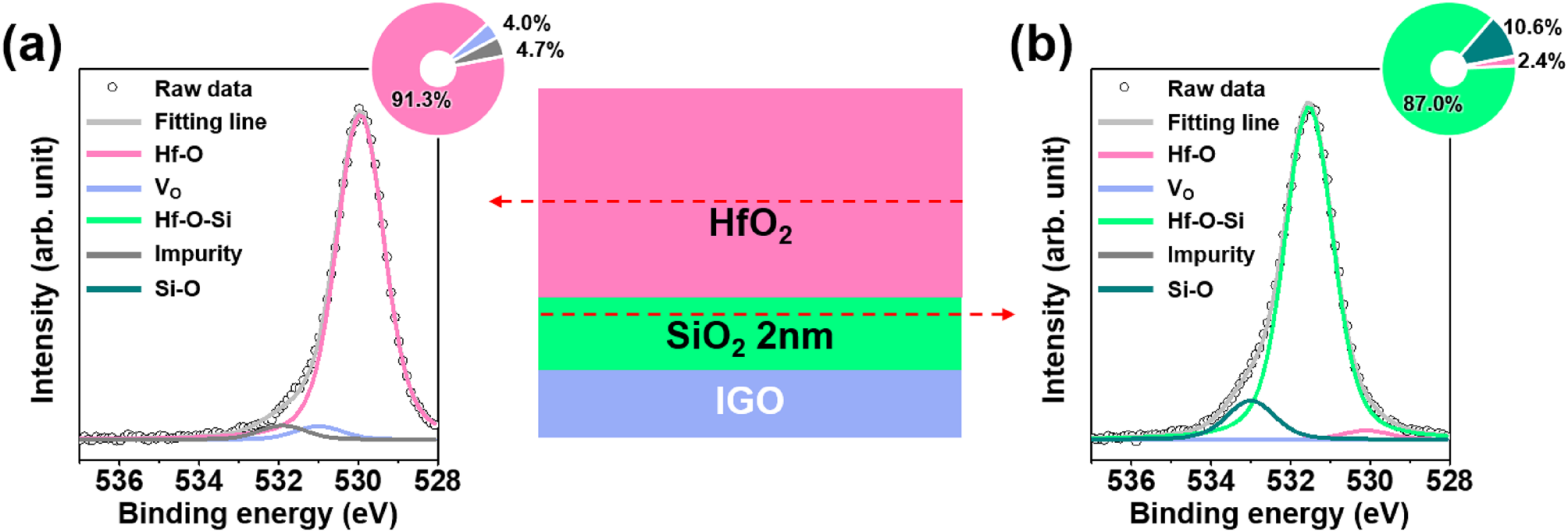
Deconvolution of the O 1*s* XPS spectra peak for (**a**) HfO_2_ and (**b**) SiO_2_ films.

Chemical states of the SiO_2_/HfO_2_ film stack were examined through XPS analysis to investigate the possible origin of RCS in the SG IGO TFTs. Figure 3a,b show the deconvoluted O 1*s* XPS spectra into five bases, corresponding to oxygen bonded to fully coordinated metal ions (Hf–O: 530.0 ± 0.1 eV, Hf–O–Si: 531.5 ± 0.1 eV, Si–O: 533.0 ± 0.1 eV) and under-coordinated oxygen related to oxygen vacancies (V_O_: 531.0 ± 0.1 eV), and impurity oxygen species (532.0 ± 0.1 eV)^27–30^. In the bulk region of HfO_2_, the Hf–O related peak is dominant (~ 91.3%). Meanwhile, hafnium silicate (Hf–O–Si) related sub-peak becomes dominant (~ 87.0%) in the SiO_2_/HfO_2_ interfacial area, indicating that the Hf element diffuses into the ultrathin SiO_2_ film during thermal annealing process. The increase in the Hf–O–Si bond can be partially attributed to the strong binding nature of Si resulting in second neighbor effect^30^. To double-check the formation of Hf–O–Si layer, Si *2p* and Hf *4f* XPS spectra were also analyzed as shown in Figure S4. In the interfacial area, the main subpeak in the Si *2p* spectra appears at 103 eV, which implies the Hf–O–Si formation^29,31^. The blue-shift of Hf *4f* XPS spectra which occurs at the interface also indicates the strong bond formation of Hf–O–Si (Figure S4c)^32^.

**Figure 4:**
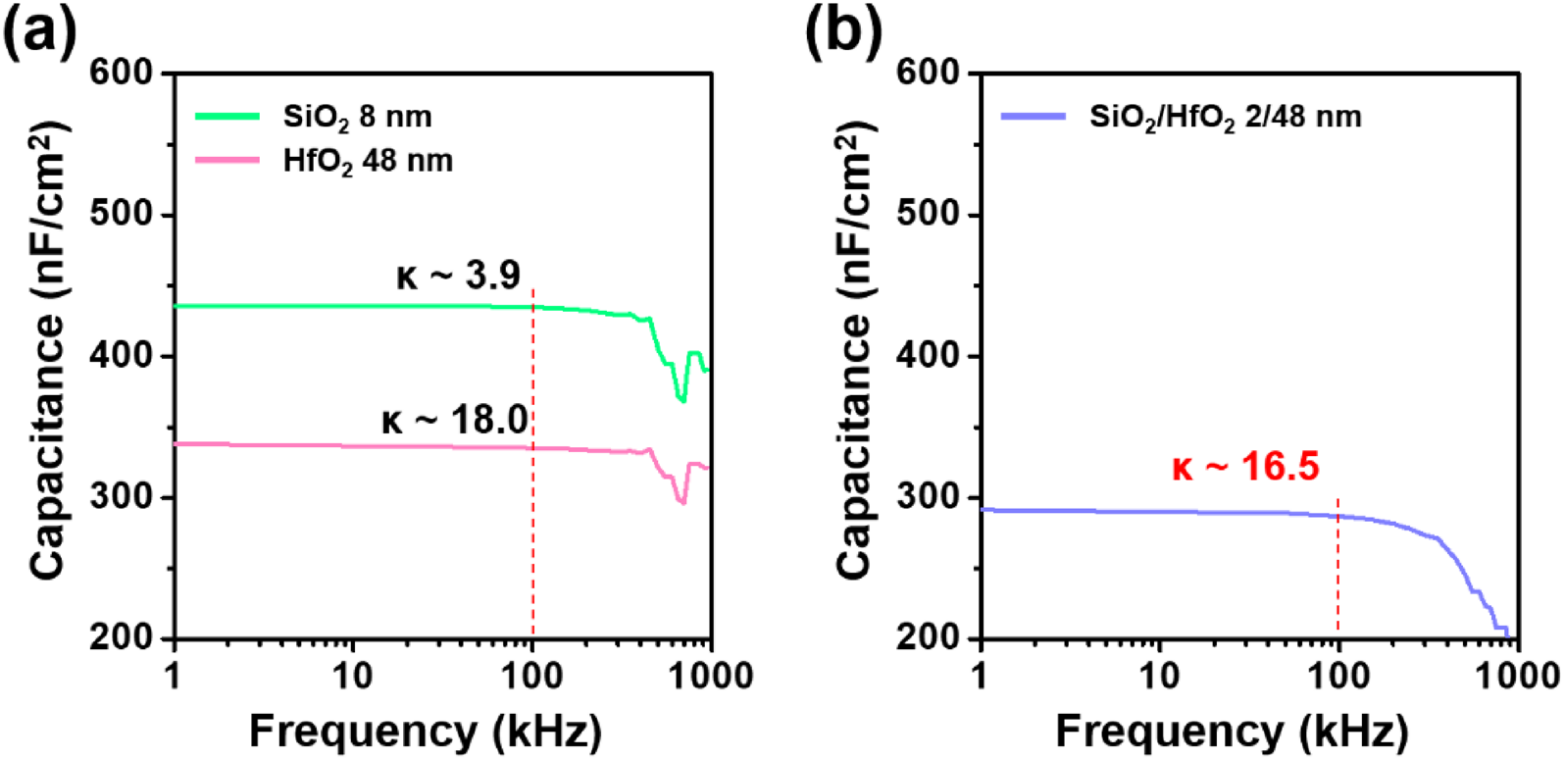
Capacitance-frequency characteristics of MIM capacitors with (**a**) SiO_2_ and HfO_2_, and (**b**) SiO_2_/HfO_2_ films.

The chemical properties are anticipated to affect their dielectric properties. The dielectric permittivity (*κ*) of SiO_2_, HfO_2_ and SiO_2_/HfO_2_ film stack was characterized by fabricating metal–insulator–metal (MIM) capacitors. The capacitances of MIM capacitors with SiO_2_ (8 nm) and HfO_2_ (48 nm) films were investigated through capacitance-frequency measurements. The *κ* values are obtained to 3.9 and 18.0 for the SiO_2_ and the HfO_2_, respectively (Fig. 4a). Using these values, the *κ* of the 2-/48-nm-thick SiO_2_/HfO_2_ can be estimated using the following equation:

4 $$\frac{1}{C}=\frac{1}{{C}_{{\text{Hf}}}}+\frac{1}{{C}_{{\text{Si}}}}\to \frac{{t}_{{\text{HS}}}}{{\kappa }_{{\text{HS}}}}=\frac{{t}_{{\text{Hf}}}}{{\kappa }_{{\text{Hf}}}}+\frac{{t}_{{\text{Si}}}}{{\kappa }_{{\text{Si}}}}$$

where *t*_HS_, *t*_Hf_, and *t*_Si_ are physical thickness of SiO_2_/HfO_2_, HfO_2_, and SiO_2_, respectively, and *κ*_HS_, *κ*_Hf_, and *κ*_Si_ are the corresponding films’ *κ* values. While the value calculated from the Eq. (4) is 15.7, the *κ*_HS_ value of SiO_2_/HfO_2_ extracted from the capacitance measurements is 16.5 (Fig. 4b). This disparity supports that the ultrathin SiO_2_ is converted to the hafnium silicate with the substantially higher permittivity (*κ* ~ 12)^29,33^ during the thermal annealing process. Reduced hysteresis and higher *µ*_FE_ for the IGO TFTs with the SiO_2_/HfO_2_ could be attributed to the less interfacial trap density of hafnium silicate than the HfO_2_ film.

**Figure 5:**
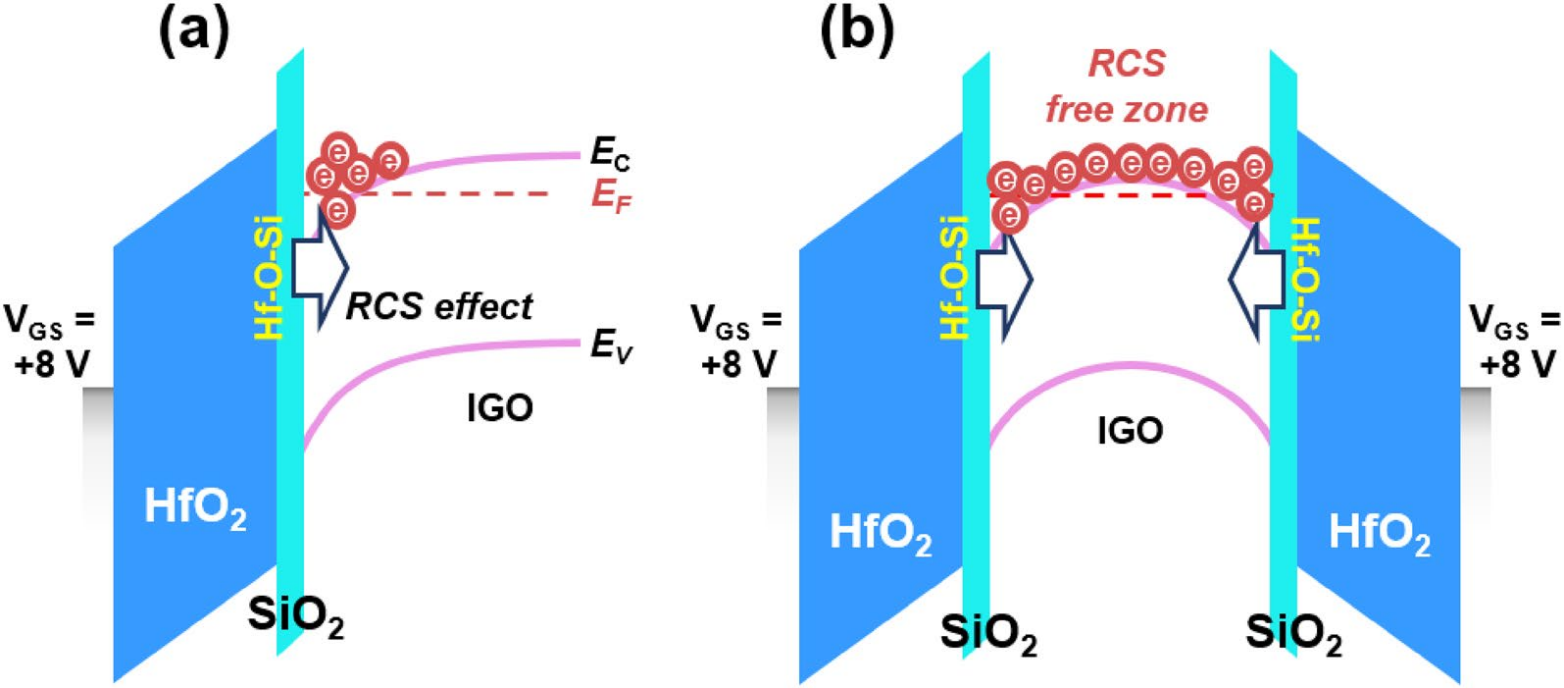
(**a**,**b**) Band diagram schematics with different driving types: (**a**) SG mode; (**b**) DG mode.

Figure 5a,b show schematic band diagrams to understand the RCS effect on electron transport during SG mode and DG mode, respectively. As the *V*_GS_ increases, the energy band of IGO with a number of subgap trap states (*N*_T_) bends downward. Note that the depth of band bending, which is often defined as the screening length, is inversely proportional to the concentration of *N*_T_^34,35^. Given that the depth of *V*_GS_-driven band bending is less than *T*_ch_, most of the free electrons exist only near the channel/gate dielectric interface (Fig. 5a). In this context, the electron transport is hampered by the RCS in the SG mode. In contrast, the free electrons can be distributed throughout the entire channel for the DG mode (Fig. 5b), because the *V*_GS_-driven band bending occurs at both sides. As a result, the RCS-free zone can be formed via the bulk accumulation in the DG IGO TFTs, improving the electron transport.

### TCAD simulation and NBTIS reliability of DG IGO TFTs

**Figure 6:**
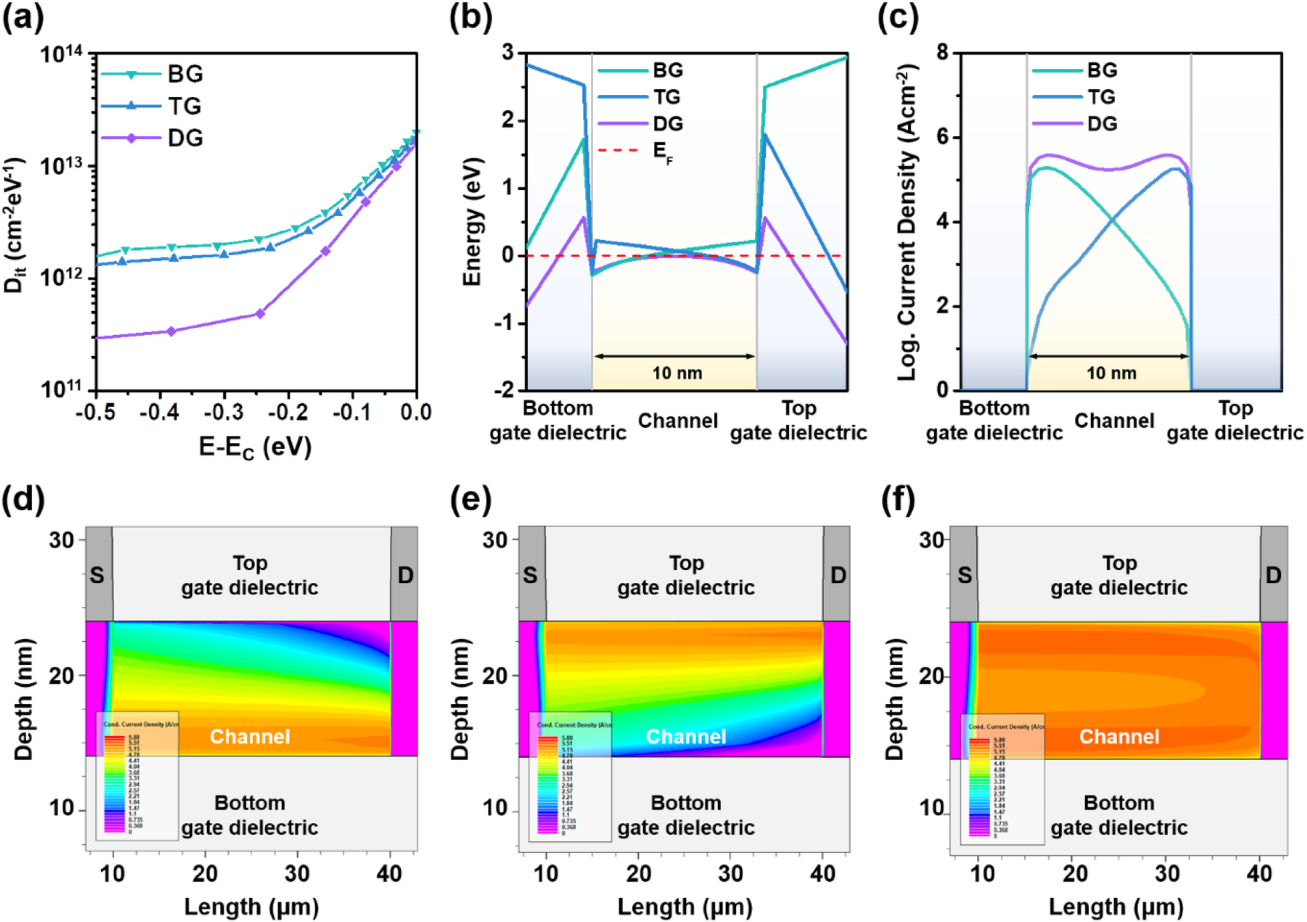
Driving mode-dependent (**a**) the *D*_it_ distributions, (**b**) the energy band diagrams, and (**c**) the depth profiles of current density. Profiles of the current density for the TFTs with (**d**) the BG, (**e**) the TG, and (**f**) the DG mode. Note that the applied *V*_GS_ and *V*_DS_ are 8 and 10 V, respectively.

Our interpretation on basis of screening length versus *T*_ch_ relation was confirmed by performing the TCAD simulation. The band structure, the density gradient quantum effect model, the remote Coulomb/phonon scattering models and the subgap density of states (DOS) were incorporated into SILVACO ATLAS. More accurate subgap density of interface states (*D*_it_) was extracted by unified subthreshold coupling factor technique^36,37^. Figure 6a shows comparison of *D*_it_ distributions for different driving mode of TFTs, which were modelled by using two exponential functions as follows:

5 $${D}_{it}\left(E\right)={N}_{tail}exp\left(-\frac{{E}_{CB} - E}{k{T}_{tail}}\right)+{N}_{deep}exp\left(-\frac{{E}_{CB} - E}{k{T}_{deep}}\right)$$

where *N*_tail_, *N*_deep_, *kT*_tail_ and *kT*_deep_ are the density of acceptor-like tail states, the acceptor-like deep states (*N*_deep_), the characteristic energy of tail states, and the characteristics energy of deep states, respectively. The parameters are summarized in Table 2. It was confirmed that the DG mode possesses a lower *D*_it_ compared to to the SG mode, even if the DG TFTs have physically two interfaces. This *D*_it_ reduction can be understood as a result reflected by the RCS-free zone as discussed earlier. Figure 6b,c show the energy level and the current density depending on the driving mode. It was confirmed that the *E*_F_ is closely located to the *E*_CB_ throughout the entire channel due to the bulk accumulation in the DG mode unlike the SG mode. As a result, the drain-current density in the DG mode is significantly higher than the SG modes, which also can be seen in Fig. 6d–f that show the cross-sectional drain-current density contours. Overall, these results demonstrate that the electron transport is greatly improved by the bulk region, the RCS-free zone, in the DG mode, which affirms the relatively minor influence of interfacial effects compared to the SG mode. Note that the simulated transfer characteristics for each driving mode is well matched with the experimental results (Figure S5). The detailed parameters of the materials and structures used in TCAD are summarized in Table S2.

**Table 2:** Electrical parameters used in the TCAD simulation for the comparison of *D*_it_ distributions.

	BG	TG	DG
*N*_tail_ [cm^−2^ eV^−1^]	1.60 × 10^13^	1.55 × 10^13^	1.50 × 10^13^
*kT*_tail_ [eV]	0.060	0.058	0.058
*N*_deep_ [cm^−2^ eV^−1^]	3.5 × 10^12^	2.5 × 10^12^	8.0 × 10^11^
*kT*_deep_ [eV]	0.45	0.45	0.25

**Figure 7:**
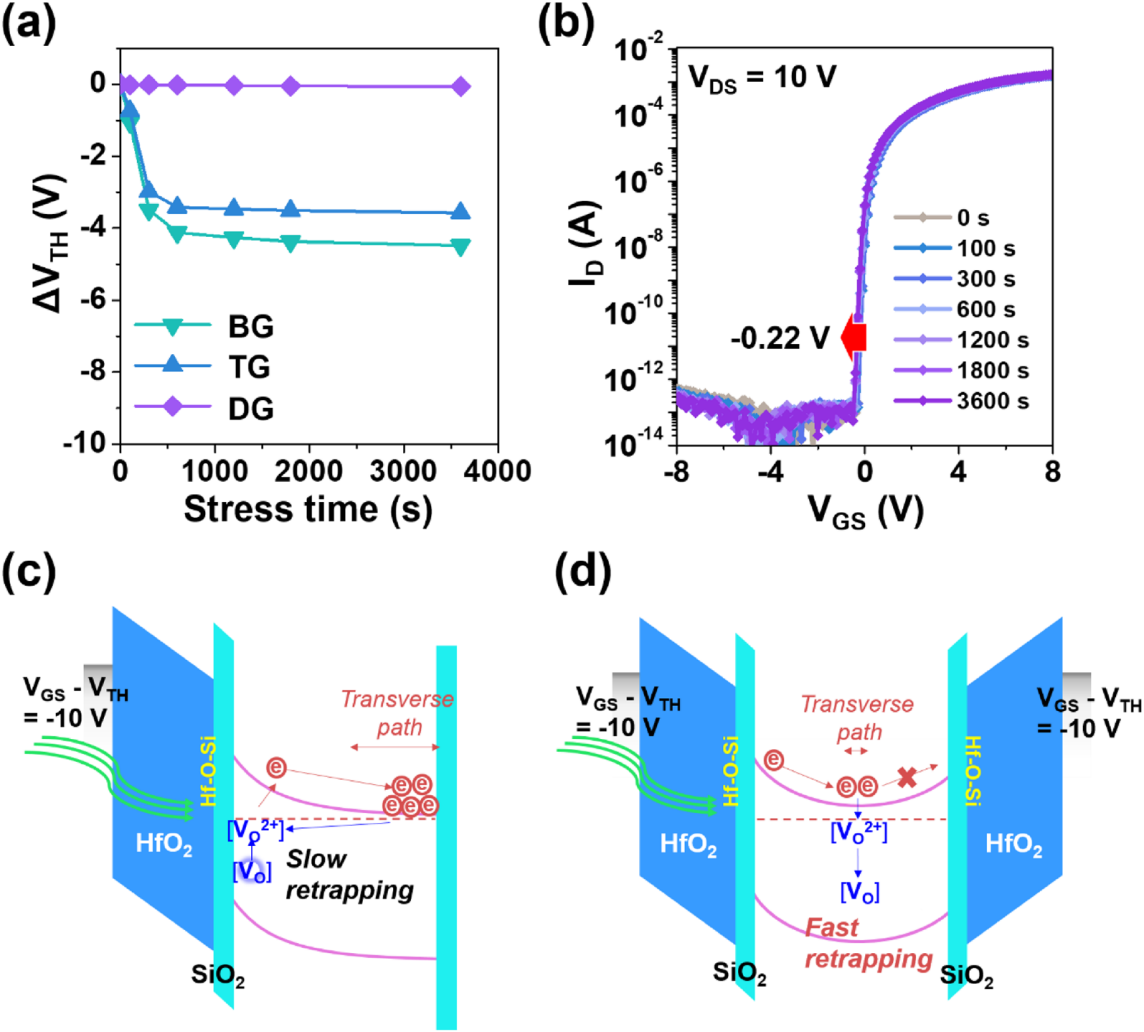
(**a**) Variations of *ΔV*_TH_ under the NBIS condition. (**b**) Stress-time dependent change of transfer characteristics of the DG IGO TFTs under the NBTIS condition. Band diagram schematics under the NB(T)IS condition for (**c**) the SG mode and (**d**) the DG mode.

Finally, negative gate bias illumination stress (NBIS) reliability was investigated for the different driving modes under the external stress conditions with an electric field of − 2 MVcm^−1^ (*V*_GS_ − *V*_TH_ =  − 10 V) and the green light illumination of 0.3 mWcm^−2^ (λ = 533 nm, 2.3 eV). The representative stress time-dependent transfer characteristics during the NBIS duration can be seen in Figure S6. It was confirmed that there are parallel shifts without involving the stretch-out of subthreshold *I*_D_ region regardless of the driving mode, suggesting that there is no noticeable defect creation during the NBIS duration. More importantly, the *V*_TH_ shift (*ΔV*_TH_) is remarkably reduced in the DG mode (Fig. 7a). While the BG (TG) IGO TFTs show an inferior NBIS instability with the *ΔV*_TH_ of − 4.5 (− 3.6) V, respectively, after 3600 s, the DG TFTs exhibit the negligible *ΔV*_TH_. Furthermore, the DG IGO TFTs reveal the outstanding reliability with the *ΔV*_TH_ of − 0.22 V even under the NBTIS with the blue light illumination of 64 *µ*Wcm^−2^ (λ = 463 nm, 2.7 eV) at 80 ℃ (Fig. 7b). These highly improved reliabilities could be related to the electron generation by the light illumination-driven transition from deep-level neutral V_O_ defects to V_O_^2+^ states^38–40^. In detail, the photo-induced V_O_^2+^ defects and free electron carriers are separated by the negative gate bias during the NBIS duration. In the SG mode, the photo electrons are repelled in the direction opposite to the biased gate electrode and accumulated. As a result, they greatly increase the electron concentration of IGO (Fig. 7c), deteriorating the NBIS reliability. Meanwhile, in the DG mode, the photo electrons cannot be accumulated at one side, which results in significant shrinkage of the electron transverse path, leading to fast re-trapping (Fig. 7d). For this reason, the electron concentration does not increase in the DG mode, showing the high reliabilities even under the light illumination.

**Figure 8:**
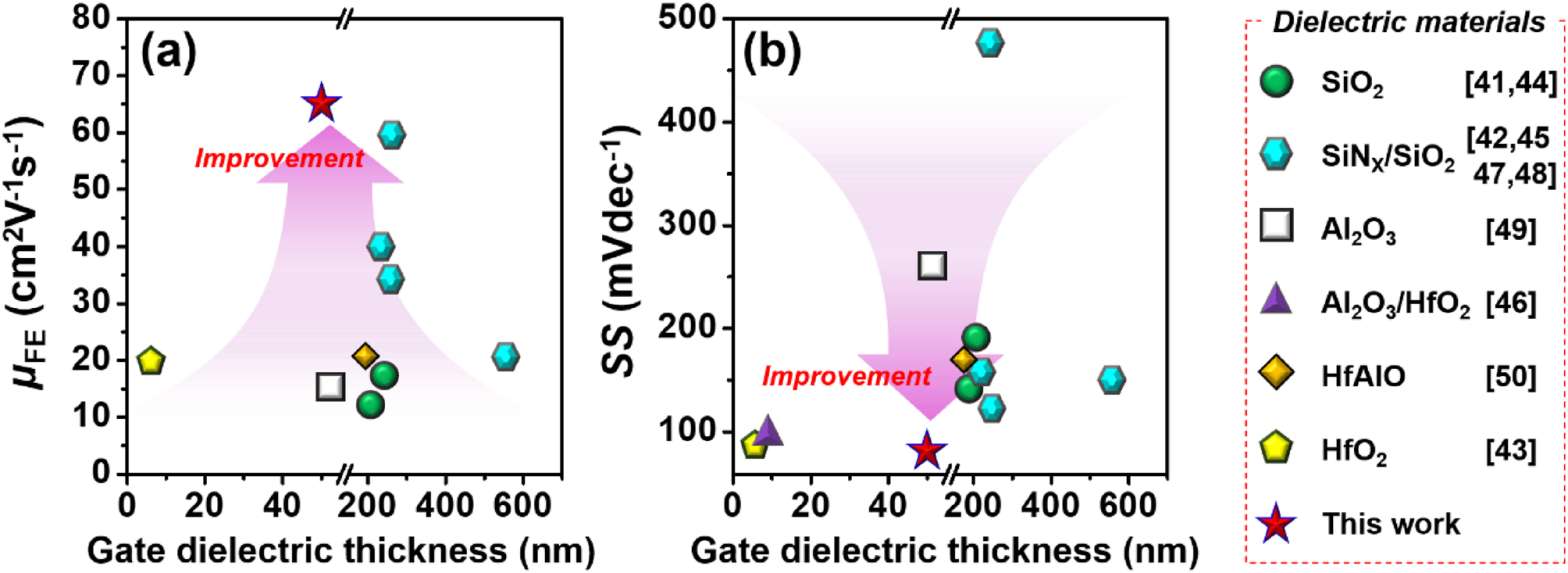
Benchmarks of (**a**) the *µ*_FE_ and (**b**) the *SS* for the DG AOS TFTs.

It is noteworthy that the electrical characteristics of DG IGO TFTs obtained in this study is comparable to state-of-the-art DG AOS TFTs. The *µ*_FE_ and *SS* values of DG AOS TFTs with different gate dielectric materials are summarized in benchmarking graphs (Fig. 8)^40–49^. These promising performances of fabricated DG IGO TFTs should be attributed to (1) the usage of a high-quality ALD-derived IGO and high-*κ* HfO_2_ dielectric films, (2) RCS-free device design on basis of bulk accumulation mode.

## Conclusion

In this study, high-performance DG IGO TFTs were fabricated using PEALD. The SG IGO TFTs with 2-/48-nm-thick SiO_2_/HfO_2_ exhibited the moderate device performances with *µ*_FE_ of 24.7 cm^2^V^−1^ s^−1^, *SS* of 110 mVdec^−1^, and *I*_ON/OFF_ of ~ 10^9^. Importantly, the DG IGO TFTs revealed greatly improved device performances with *µ*_FE_ of 65.2 ± 2.3 cm^2^V^−1^ s^−1^, *SS* of 65 ± 1 mVdec^−1^, and *I*_ON/OFF_ of ~ 10^10^. This disparity can originate from the bulk accumulation, the formation of RCS-free zone, because the electron transport via the RCS-free zone helps the *E*_F_ reach the *E*_CB_ rapidly. This elucidation was supported through TCAD simulation. Finally, it was confirmed that the DG IGO TFTs show the photo-bias stability much superior to the SG IGO TFTs, which can be attributed to the fast re-trapping of the photo electrons released from deep-level V_O_ defects. It should be emphasized that even if this study focuses on effects of the DG structure on the IGO TFTs, the obtained results can be applicable to more advanced multi-gate AOS TFTs.

### Supplementary Information

Supplementary Information.

## Data Availability

The datasets used and/or analyzed during the current study available from the corresponding author on reasonable request. Correspondence and requests for materials should be addressed to J.K.J. (email: jkjeong@hanyang.ac.kr).

## References

[1] Nomura K, Ohta H, Takagi A, Kamiya T, Hirano M, Hosono H (2004). Room-temperature fabrication of transparent flexible thin-film transistors using amorphous oxide semiconductors. Nature.

[2] Kim BK, On N, Choi CH, Kim MK, Kang S, Lim JH, Jeong JK (2021). Polycrystalline indium gallium tin oxide thin-film transistors with high mobility exceeding 100 cm^2^/Vs. IEEE Electron Device Lett..

[3] Choi CH, Kim T, Ueda S, Shiah Y-S, Hosono H, Jeong JK (2021). High-performance indium gallium tin oxide transistors with an Al_2_O_3_ gate dielectric deposited by atomic layer deposition at a low temperature of 150 °C: Roles of hydrogen and excess oxygen in the Al_2_O_3_ dielectric film. ACS Appl. Mater. Interfaces.

[4] Cho MH, Choi CH, Seul HJ, Cho HC, Jeong JK (2021). Achieving a low-voltage, high-mobility IGZO transistor through an ALD-derived bilayer channel and a hafnia-based gate dielectric. ACS Appl. Mater. Interfaces.

[5] Seul HJ, Cho JH, Hur JS, Cho MH, Cho MH, Ryu MT, Jeong JK (2022). Improvement in carrier mobility through band-gap engineering in atomic-layer-deposited In-Ga-Zn-O stacks. J. Alloys Compd..

[6] Kim MJ, Park HJ, Yoo S, Cho MH, Jeong JK (2022). Effect of channel thickness on performance of ultrathin body IGZO field-effect transistors. IEEE Trans. Electron Devices.

[7] Lee J, Choi CH, Kim T, Hur J, Kim MJ, Kim EH, Lim JH, Kang Y, Jeong JK (2022). Hydrogen-doping-enabled boosting of the carrier mobility and stability in amorphous IGZTO transistors. ACS Appl. Mater. Interfaces.

[8] Kim T, Choi CH, Hur JS, Ha D, Kuh BJ, Kim Y, Cho MH, Kim S, Jeong JK (2023). Progress, challenges, and opportunities in oxide semiconductor devices: A key building block for application from display backplanes to 3D integrated semiconductor chips. Adv. Mater..

[9] Cho MH, Choi CH, Kim MJ, Hur JS, Kim T, Jeong JK (2022). Comparative study of atomic layer deposited indium-based oxide transistors with a fermi energy level-engineered heterojunction structure channel through a cation combinatorial approach. ACS Appl. Mater. Interfaces.

[10] Park HY, Kim T, Kim MJ, Lee H, Lim JH, Jeong JK (2022). Improvement in performance of indium gallium oxide thin film transistor via oxygen mediated crystallization at a low temperature of 200 °C. Ceram. Int..

[11] Cho MH, Choi CH, Jeong JK (2023). High-performance indium-based oxide transistors with multiple channels through nanolaminate structure fabricated by plasma-enhanced atomic layer deposition. ACS Appl. Mater. Interfaces.

[12] Kim G-B, On N, Kim T, Choi CH, Hur JS, Lim JH, Jeong JK (2023). High mobility IZTO thin-film transistors based on spinel phase formation at low temperature through a catalytic chemical reaction. Small Methods.

[13] Na J-S, Hong S-K, Kwong O-K (2019). A 4410-ppi resolution pixel circuit for high luminance uniformity of OLEDoS microdisplays. IEEE J. Electron Devices Soc..

[14] Chen T-L, Huang K-C, Lin H-Y, Chou CH, Lin HH, Liu CW (2013). Enhanced current drive of double-gate a-IGZO thin-film transistors. IEEE Electron Device Lett..

[15] Mativenga M, An S, Jang J (2013). Bulk accumulation a-IGZO TFT for high current and turn-on voltage uniformity. IEEE Electron Device Lett..

[16] Park J, Park S, Jang JT, Choi S-J, Kim DM, Bae J-H, Shin HJ, Jeong YS, Bae JU, Oh CH, Kim C, Kim DH (2022). Effect of positive bias stress on the back-gate voltage-modulated threshold voltage in double-gate amorphous InGaZnO thin-film transistors. IEEE Electron Device Lett..

[17] More Moore-International Roadmap for Devices and Systems (IRDS), Available: https://irds.ieee.org/images/files/pdf/2022/2022IRDS_MM.pdf (2022).

[18] Herrera, F. Á., Mattausch, M., Iizuka, T., Kilkuchihara, H., Hirano, Y. & Mattausch, H. J. Modeling of short-channel effect on multi-gate MOSFETs for circuit simulation. In *2020 International Symposium on Devices, Circuits and Systems (ISDCS)*, 1−4. 10.1109/ISDCS49393.2020.9263000 (2020).

[19] Ferain I, Colinge CA, Colinge J-P (2011). Multigate transistors as the future of classical metal-oxide-semiconductor field-effect transistors. Nature.

[20] Liu Y, Duan X, Shin H-J, Park S, Huang Y, Duan X (2021). Promises and prospects of two-dimensional transistors. Nature.

[21] Cho MH, Seol H, Song A, Choi S, Song Y, Yun PS, Chung K-B, Bae JU, Park K-S, Jeong JK (2019). Comparative study on performance of IGZO transistors with sputtered and atomic layer deposited channel layer. IEEE Trans. Electron Devices.

[22] Kim H-R, Kim G-H, Seong N-J, Choi K-J, Kim S-K, Yoon S-M (2020). Comparative studies on vertical-channel charge-trap memory thin-film transistors using In-Ga-Zn-O active channels deposited by sputtering and atomic layer deposition. Nanotechnology.

[23] Duan X, Huang K, Feng J, Niu J, Qin H, Yin S, Jiao G, Leonelli D, Zhao X, Wang Z, Jing W, Wang Z, Wu Y, Xu J, Chen Q, Chuai X, Lu C, Wang W, Yang G, Geng D, Li L (2022). Novel VERTICAL CHANNEL-ALL-AROUND (CAA) In-Ga-Zn-O FET for 2T0C-DRAM with high density beyond 4F2 by monolithic stacking. IEEE Trans. Electron Devices.

[24] Bai Z, Gong T, Duan X, Wang J, Xiao K, Geng D, Li L (2022). Low frequency noise of channel-all-around (CAA) InGaZnO field effect transistors. IEEE Electron Device Lett..

[25] Yan R-H, Ourmazd A, Lee KF (1992). Scaling the Si MOSFET: From bulk to SOI to bulk. IEEE Trans. Electron Devices.

[26] Park J-S, Jeong JK, Mo YG, Kim S (2009). Impact of high-*κ* TiOx dielectric on device performance of indium-gallium-zinc oxide transistors. Appl. Phys. Lett..

[27] Zhang Y, He G, Wang W, Yang B, Zhang C, Xia Y (2020). Aqueous-solution-driven HfGdOx gate dielectrics for low-voltage-operated a-InGaZnO transistors and inverter circuits. J. Mater. Sci. Technol..

[28] Avis C, Kim YG, Jang J (2012). Solution processed hafnium oxide as a gate dielectric for low-voltage oxide thin-film transistors. J. Mater. Chem..

[29] Lee S, Yun D-J, Rhee S-W, Yong J (2009). Atomic layer deposition of Hafnium silicate film for high mobility pentacene thin film transistor applications. J. Mater. Chem..

[30] Wang H, Hg KL, Zhan N, Poon MC, Kok CW (2004). Interface bonding structure of hafnium oxide prepared by direct sputtering of hafnium in oxygen. J. Vac. Sci. Technol. B.

[31] Torchynska T, Macotela LGV, Khomenkova L, Gourbilleau F, Rojas LL (2020). Annealing impact on emission and phase varying of Nd-doped Si-rich-HfO_2_ films prepared by RF magnetron sputtering. J. Mater. Sci. Mater. Electron..

[32] Wang H, Wu P, Li XF, Chen S, Zhang SP, Song BB (2011). Study of reactions between HfO_2_ and Si in thin films with precise identification of chemical states by XPS. Appl. Surf. Sci..

[33] Lee J-C, Oh S-J, Cho M, Hwang CS, Jung R (2004). Chemical structure of the interface in ultrathin HfO_2_/Si films. Appl. Phys. Lett..

[34] Park J-S, Jeong JK, Chung H-J, Mo Y-G, Kim HD (2008). Electronic transport properties of amorphous indium-gallium-zinc oxide semiconductor upon exposure to water. Appl. Phys. Lett..

[35] Zhou X, Shao Y, Zhang L, Xiao X, Han D, Wang Y, Zhang S (2017). Oxygen adsorption effect of amorphous InGaZnO thin-film transistors. IEEE Electron Device Lett..

[36] Jun S, Jo C, Bae H, Choi H, Kim DH, Kim DM (2013). Unified subthreshold coupling factor technique for surface potential and subgap density-of-states in amorphous thin film transistors. IEEE Electron Device Lett..

[37] Lee S, Nathan A, Ye Y, Guo Y, Robertson J (2015). Localized tatil states and electron mobility in amorphous ZnON thin film transistors. Sci. Rep..

[38] Ji KH, Kim J-I, Jung HY, Park SY, Choi R, Mo YG, Jeong JK (2011). Comprehensive studies of the degradation mechanism in amorphous InGaZnO transistors by the negative bias illumination stress. Microelectron. Eng..

[39] Song JH, On N, Du B, Kim HD, Jeong JK (2016). Dynamics of threshold voltage instability in IGZO TFTs: Impact of high pressurized oxygen treatment on the activation energy barrier. IEEE Trans. Electron Devices.

[40] Mativenga M, Haque F, Billah MM, Um JG (2021). Origin of light instability in amorphous IGZO thin-film transistors and its suppression. Sci. Rep..

[41] Lee J, Kim D, Lee S, Cho J, Park H, Jang J (2019). High field effect mobility, amorphous In-Ga-Sn-O thin-film transistor with no effect of negative bias illumination stress. IEEE Electron Device Lett..

[42] Chakraborty W, Ye H, Grisafe B, Lightcap I, Datta S (2020). Low temperature budget (<250 ℃) dual-gate amorphous indium tungsten oxide (IWO) thin-film transistor for monolithic 3−D integration. IEEE Trans. Electron Devices.

[43] Rabbi MH, Billah MM, Siddik AB, Lee S, Lee J, Jang J (2020). Extremely stable dual gate coplanar amorphous InGaZnO thin film transistor with split active layer by N_2_O annealing. IEEE Electron Device Lett..

[44] Her J, Min WK, Shin CS, Jeong H, Park JK (2022). A new pixel circuit with selectively synchronized dual-gated IGZTO TFTs for AMOLED displays. IEEE Trans. Electron Devices.

[45] Lu, W., Zhu, Z., Chen, K., Liu, M., Kang, B.-M., Duan, X., Niu, J., Liao, F., Dan, W., Wu, X.-S., Son, J., Xiao, D.-Y., Wang, G.-L., Yoo, A., Cao, K.-Y., Geng, D., Lu, N., Yang, G., Zhao, C., Li, L. & Liu, M. First demonstration of dual-gate IGZO 2T0C DRAM with novel read operation, one bit line in single cell, I_ON_ = 1500 µA/µm@V_DS_ = 1 V and retention time>300s. In *Proceedings of the 2022 International Electron Devices Meeting (IEDM)*, 26.4.1−26.4.4. 10.1109/IEDM45625.2022.10019488 (2022).

[46] Liu W-S, Hsu C-H, Jiang Y, Lai Y-C, Kuo H-C (2022). Improving device characteristics of dual-gate IGZO thin-film transistors with Ar-O_2_ mixed plasma treatment and rapid thermal annealing. Membranes.

[47] Priyadarshi S, Billah MM, Kim H, Rabbi MH, Urmi SS, Lee S, Jang J (2022). High-performance dual gate amorphous InGaZnO thin film transistor with top gate to drain offset. IEEE Electron Device Lett..

[48] Yang J, Park H, Kim B, Cho Y-H, Park S-HK (2022). Active-matrix micro-light-emitting diode displays driven by monolithically integrated dual-gate oxide thin-film transistors. J. Mater. Chem. C.

[49] Kong H, Cho K, Kang M, Kim J, Lee S, Lim J, Kim S (2023). Effect of dual gating on electrical characteristics of amorphous indium–tin–gallium–zinc-oxide TFTs. Electron. Lett..

