# Supplementary material for: Double-gate structure enabling remote Coulomb scattering-free transport in atomic-layer-deposited IGO thin-film transistors with HfO2 gate dielectric through insertion of SiO2 interlayer
Source: Sci Rep. 2024 Apr 1;14:7623. doi: 10.1038/s41598-024-58330-1 (PMC10984952; doi:10.1038/s41598-024-58330-1)
Supplement: Supplementary file 1 — Supplementary Information. [file 41598_2024_58330_MOESM1_ESM.docx]

**Supplementary Information**

**Double-gate structure enabling remote Coulomb scattering-free transport in atomic-layer-deposited IGO thin-film transistors with HfO_2_ gate dielectric through insertion of SiO_2_ interlayer**

Cheol Hee Choi,^1,†^ Taikyu Kim,^2,†^ Min Jae Kim^1^, Gwang-Bok Kim^1^, Jeong Eun Oh^1^, and Jae Kyeong Jeong^1,*^

^1^Department of Electronic Engineering, Hanyang University, Seoul 04763, Republic of Korea

^2^Electronic Materials Research Center, Korea Institute of Science and Technology, Seoul 02792, Republic of Korea

Corresponding author: J. K. Jeong (jkjeong1@hanyang.ac.kr).


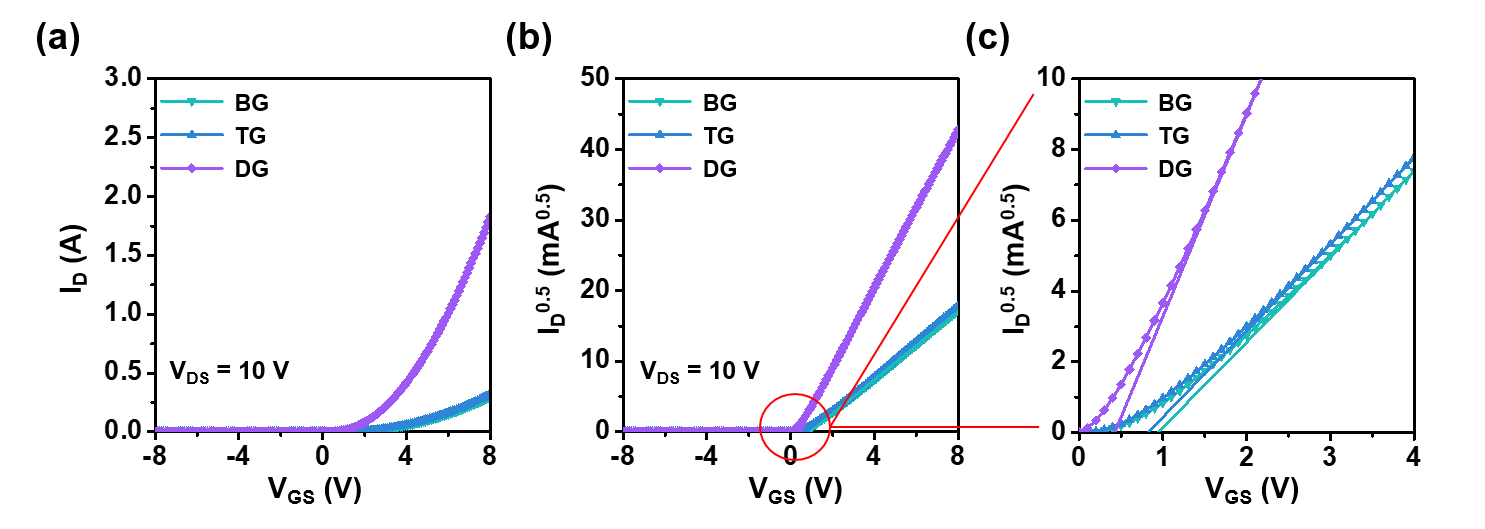


Figure S1. (a) Linear *I*_D_-*V*_GS_ characteristics of BG, TG and DG mode IGO TFTs. (b) *V*_TH_ extraction using linear extrapolation method, which was called as second-derivative method. (c) An extended graph of (b).


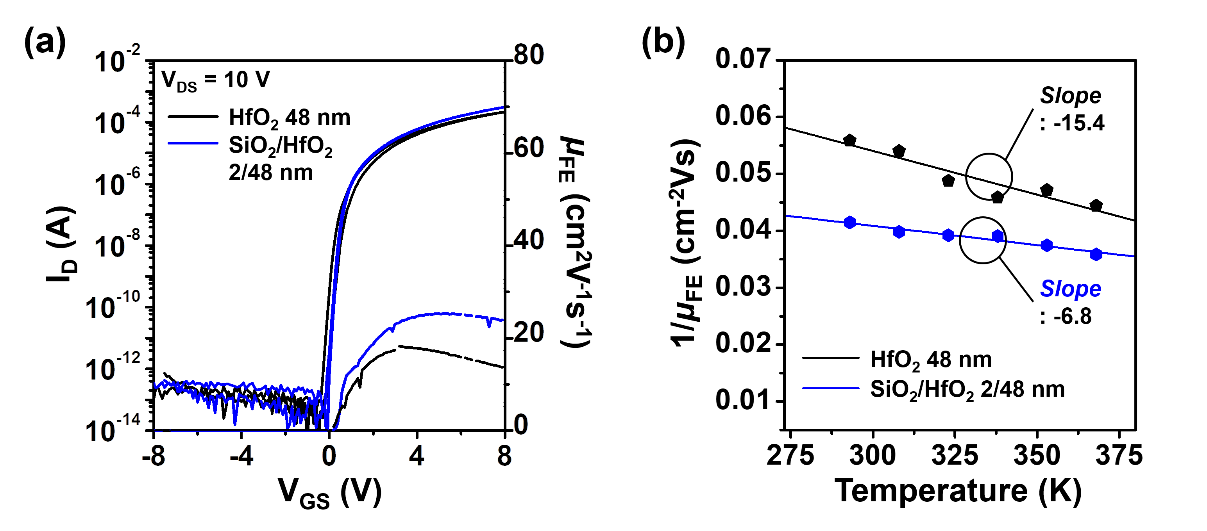


Figure S2. (a) Representative transfer characteristics of IGO TFTs with a different gate dielectric layer. (b) Extracted 1/*µ*_FE_-*T* characteristics.

**Table S1.** Summary of electrical parameters of the single gate IGO TFTs with different gate dielectric.

|  | HfO_2_ 48 nm | SiO_2_/HfO_2_ 2/48 nm | |
| --- | --- | --- | --- |
| *μ*_FE_ [cm^2^V^-1^s^-1^] | 18.1 ± 1 | 24.7 ± 0.7 | |
| *SS* [mVdec^-1^] | 130 ± 5 | 110 ± 5 | |
| *V*_TH_ [V] | 0.22 ± 0.2 | 0.40 ± 0.1 |  |
| *I*_ON/OFF_ | ~10^9^ | ~10^9^ | |
| Hysteresis [mV] | ~300 | ~50 | |
| Slope value | −15.4 | −6.8 | |


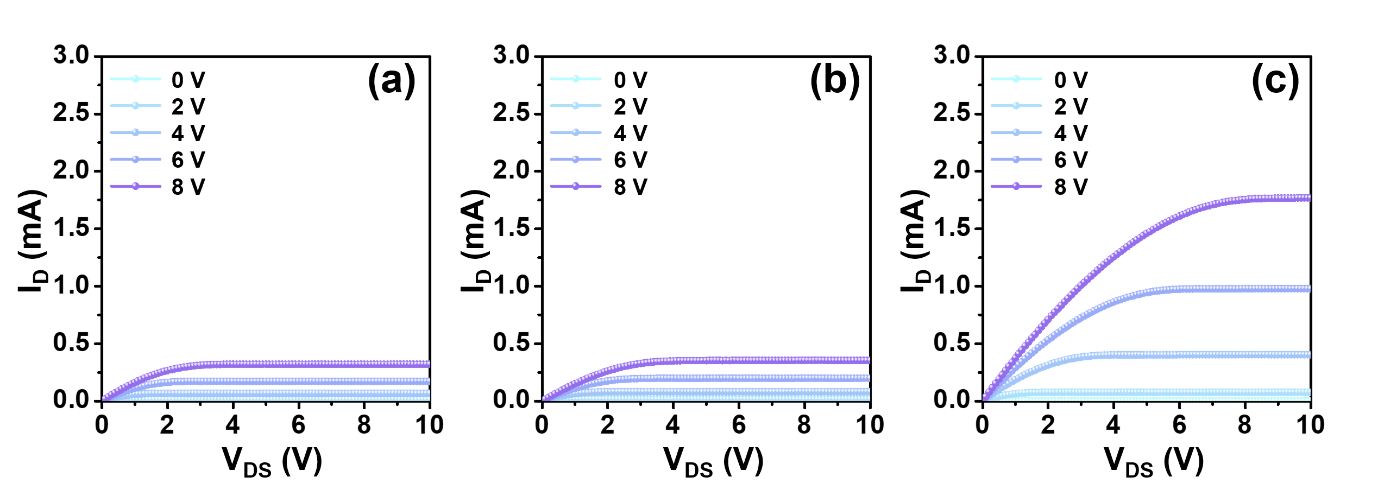


**Figure S3.** Representative output characteristics of (a) BG, (b) TG, and (c) DG IGO TFTs, respectively. The *V*_GS_ is varied from 0 to 8 V.


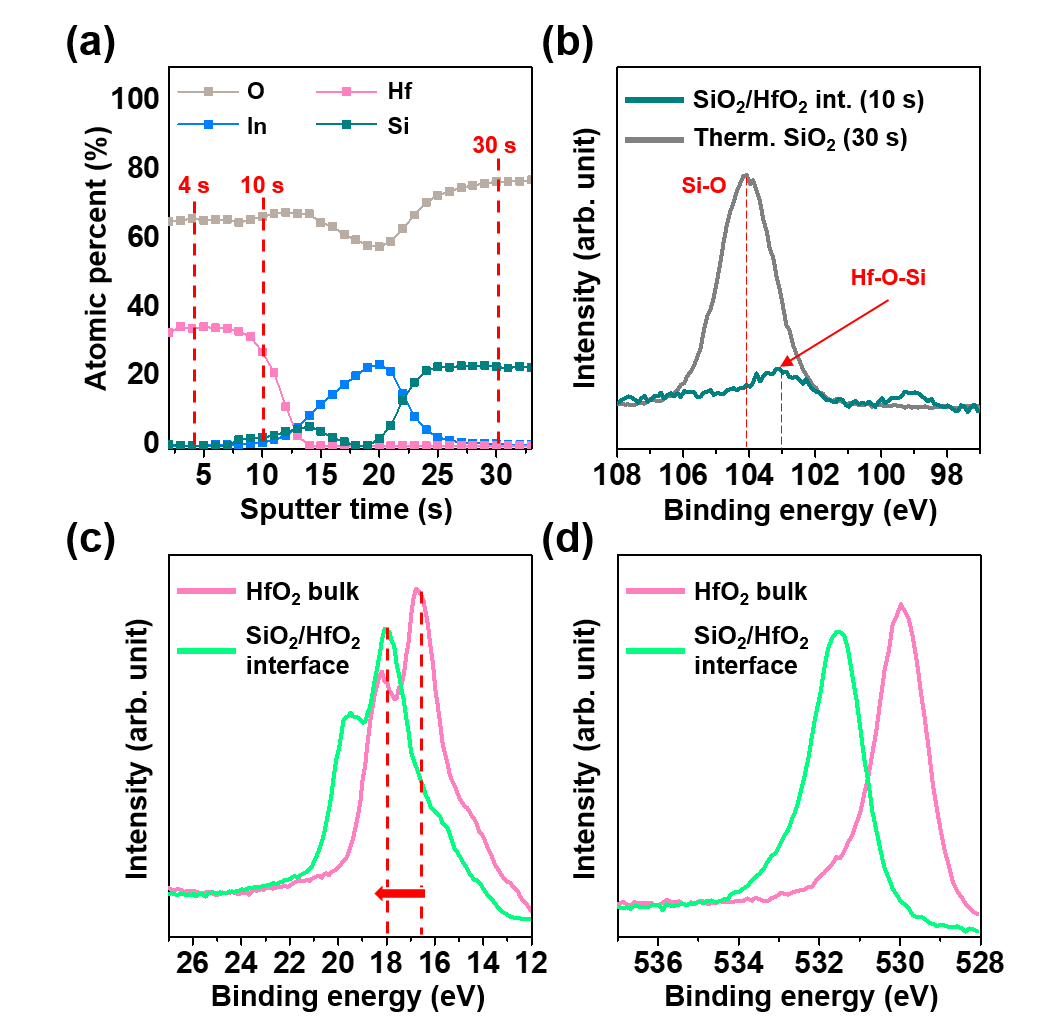


**Figure S4.** (a) XPS depth profiles of HfO_2_/SiO_2_/IGO grown on the SiO_2_ (buffer) substrate. (b) Si *2p*, (c) Hf *4f*, and (d) O *1s* XPS spectra. The Si *2p* XPS spectra for the thermal SiO_2_ (buffer layer) is present for comparison (see Fig. S4(b)). While a main peak is observed at 104 eV for the thermal SiO_2_ film, the Hf-O-Si related subpeak located at 103 eV appears for the ultrathin SiO_2_ film inserted between the HfO_2_ and IGO films. The blue-shifts of (c) Hf *4f* and (d) O *1s* XPS spectra also imply the strong bond formation of Hf-O-Si.


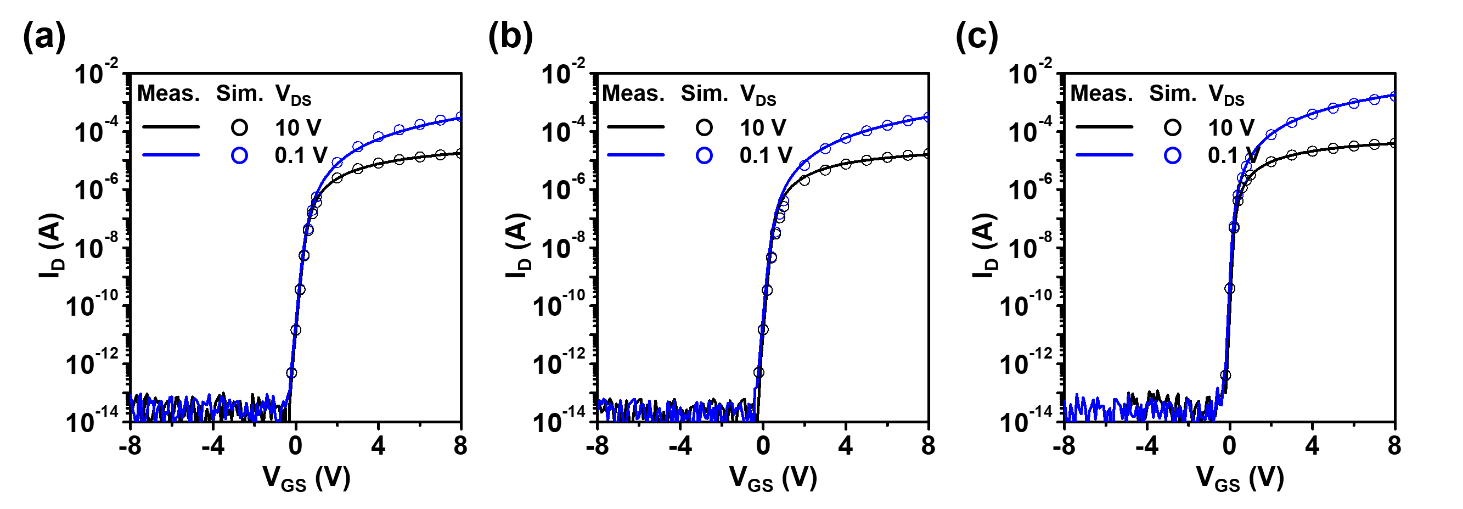


**Figure S5.** Comparison of measured and simulated transfer characteristics for (a) the BG, (b) the TG and (c) the DG IGO TFTs.

**Table S2.** Summary of material and structural parameters used in the TCAD.

| **Parameter Symbol** | **Definition** | **Value** | **Unit** |
| --- | --- | --- | --- |
| **E_g.IGO_** | Bandgap energy of IGO | 3.2 | eV |
| **T_IGO_** | Thickness of IGO | 10 | nm |
| **N_CB.IGO_** | Density of conduction band states of IGO | 5 x 10^18^ | cm^− 3^ |
| **μ_IGO_** | Electron mobility of IGO | 70 | cm^2^V^−1^s^−1^ |
| **T_EOT_** | Equivalent oxide thickness | 12 | nm |


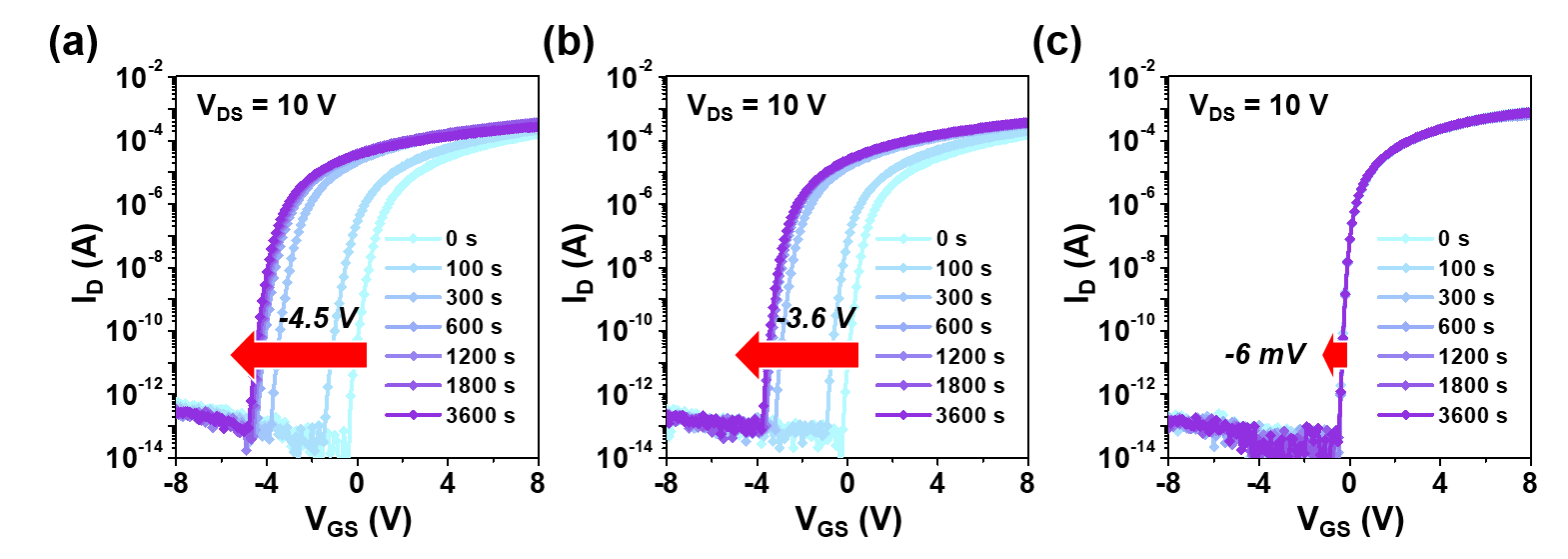


**Figure S6.** Evolution in transfer characteristics as a function of stress time under NBIS duration in green light illumination of (a) the BG, (b) the TG, and (c) the DG mode of IGO TFTs.
